# Our Contemporaries

**Published:** 1914-02

**Authors:** 


### OUR CONTEMPORARIES

---

The *Medical Economist* of December, 1913, contains a rather caustic article by Maxwell L. Volk of New York, on hospital appointments, interne and staff. It would seem that a common sense code on this matter would be about as follows: A private hospital has a right of choice based on private preferences; a hospital supported by any particular race, denomination or organization, has a right to select accordingly; a public hospital, including one supported by local governments and by public charity, is a public trust and we dislike to think that anyone sufficiently prominent to be on its staff would be actuated by favoritism.

Burton J. Hendrick, in the January *McClure's* presents an interesting discussion of the new medical ethics. He thinks that the difficult problem of privileged communications should be decided according to the results on potential victims, rather than by the obligation of the physician to an individual patient. We question whether the candor which he advocates in telling patients of incurable conditions ought always to be practiced. He has rather magnified the importance which the profession applies to mere etiquette, although most etiquette is based on common sense. The prevalence of fee splitting is greatly exaggerated.

The editorial in the *Jour. of the A. M. A.*, January 10, on deaths of physicians, deserves careful reading. Especially deplorable are the considerable number of deaths by suicide and homicide.

The *Medical Council* of January refers to abstracts in medical journals as the "medical silo." But ensilage is pretty good fodder.

The *International Hospital Record,* December 17, 1913, calls attention editorially to the advisability of giving medical students experience in nursing in hospital wards.

**The Running Expenses of a City Doctor.**

In view of the Income Tax it seems apropos to make a rough estimate of the running expenses of an average city doctor. Such an estimate as we are going to make refers especially to cities in the South where the great majority of doctors have an office establishment which is separate and distinct from the home. The automobile expenses we consider a *sine qua non* in any city of 75,000 inhabitants or more, except in the very large cities like Chicago, New York, Buffalo and others where the transportation facilities are such that nearly any home can be reached by some of the numerous methods of public transport.

| | |
|---|---|
| Automobile upkeep | $300 00 |
| Telephone | 60 00 |
| Office girl | 144 00 |
| Office rent | 240 00 |
| County and State Society, including journal | 7 00 |
| American Medical Ass'n, including journal | 5 00 |
| One good journal | 5 00 |
| Physician's Liability Insurance | 20 00 |
| Books | 30 00 |
| Medicines and Reagents for office | 10 00 |
| Dressings and new instruments | 25 00 |
| | $846.00 |

—*Nashville Medical Journal.*

